# Successful treatment of acute tubulointerstitial nephritis probably due to Benikoji CholesteHelp^®^, a supplement containing red yeast rice

**DOI:** 10.1007/s13730-024-00897-9

**Published:** 2024-06-17

**Authors:** Masayuki Maiguma, Masao Kihara, Maki Hamaguchi, Takashi Kobayashi, Koshi Yamada, Miyuki Takagi, Harumi Saeki, Tomohito Gohda, Yusuke Suzuki

**Affiliations:** 1 Department of Nephrology, Faculty of Medicine, Juntendo University, 2-1-1, Hongo, Bunkyo‑Ku, Tokyo, 113‑8421 Japan; 2 Department of Human Pathology, Faculty of Medicine, Juntendo University, 2-1-1, Hongo, Bunkyo‑Ku, Tokyo, 113‑8421 Japan

**Keywords:** Benikoji CholesteHelp®, Red yeast rice, Tubulointerstitial nephritis, Drug induced

## Abstract

Red yeast rice has been used to produce alcoholic beverages and various fermented foods especially in East Asia. Since around March 2024, there have been many cases of kidney dysfunction in people who have taken certain supplements containing red yeast rice in Japan. We experienced a case of acute kidney injuries induced after taking a supplement containing red yeast rice. A 58-year-old woman was admitted to our hospital due to renal dysfunction suspected to be caused by taking the supplement Benikoji CholesteHelp^®^, which contains red yeast rice. With elevations of urinary tubular injury markers such as urinary β2-microglobulin and *N*-acetyl-β-D-glucosaminidase, serum creatinine levels were elevated up to 2.75 mg/dL. A kidney biopsy revealed a diagnosis of tubulointerstitial nephritis with lymphocytic infiltration of the interstitium, tubular atrophy, and interstitial fibrotic changes. After discontinuation of the supplement and initiation of the prednisolone treatment, renal dysfunction rapidly improved. The course of this case suggests tubular damage caused by the supplements containing red yeast rice. For early diagnosis and treatment, it should be noted that even what are regarded as nutritional health supplements can cause renal dysfunction.

## Introduction

Supplements are often used for health maintenance and improvement. They are readily accessible at pharmacies and convenience stores. In recent years, the consumption of supplements has been spreading along with the increase in health consciousness in Japan. Dietary supplements contain various additives. However, the consumption of them can sometimes cause health problems for individuals. Non-steroidal anti-inflammatory drugs and antibiotic drugs are often implicated as a cause of drug-induced kidney injury, but it is important to note that supplements can also be a contributing factor.

## Case report

A 58-year-old woman with no history of kidney disease was admitted to our hospital for examination and treatment of kidney dysfunction. She had annual medical check-ups and had a history of dyslipidemia, but was not taking any medication. She had no other significant medical history, family history, or allergies.

About 6 weeks prior to admission, she had started taking cholesterol-lowering supplements containing red yeast rice (Benikoji CholesteHelp^®^). She took the prescribed dosage of three tablets per day (3 mg of rice red yeast polyketide) and noticed mild fever (37 ℃), nausea, and appetite loss 3 weeks after starting the medication. Therefore, she discontinued the medication 1 month later. After that, she visited a local clinic, where blood tests revealed kidney dysfunction with a serum creatinine (Cr) level of 2.75 mg/dL, and she was referred to our hospital. The examinations in our hospital revealed kidney dysfunction with the levels of serum Cr 1.57 mg/dL, urinary β2-microglobulin (β2MG) 126,473 μg/L, and urinary *N*-acetyl-β-D-glucosaminidase (NAG) 71.7 IU/L.

**Fig. 1:**
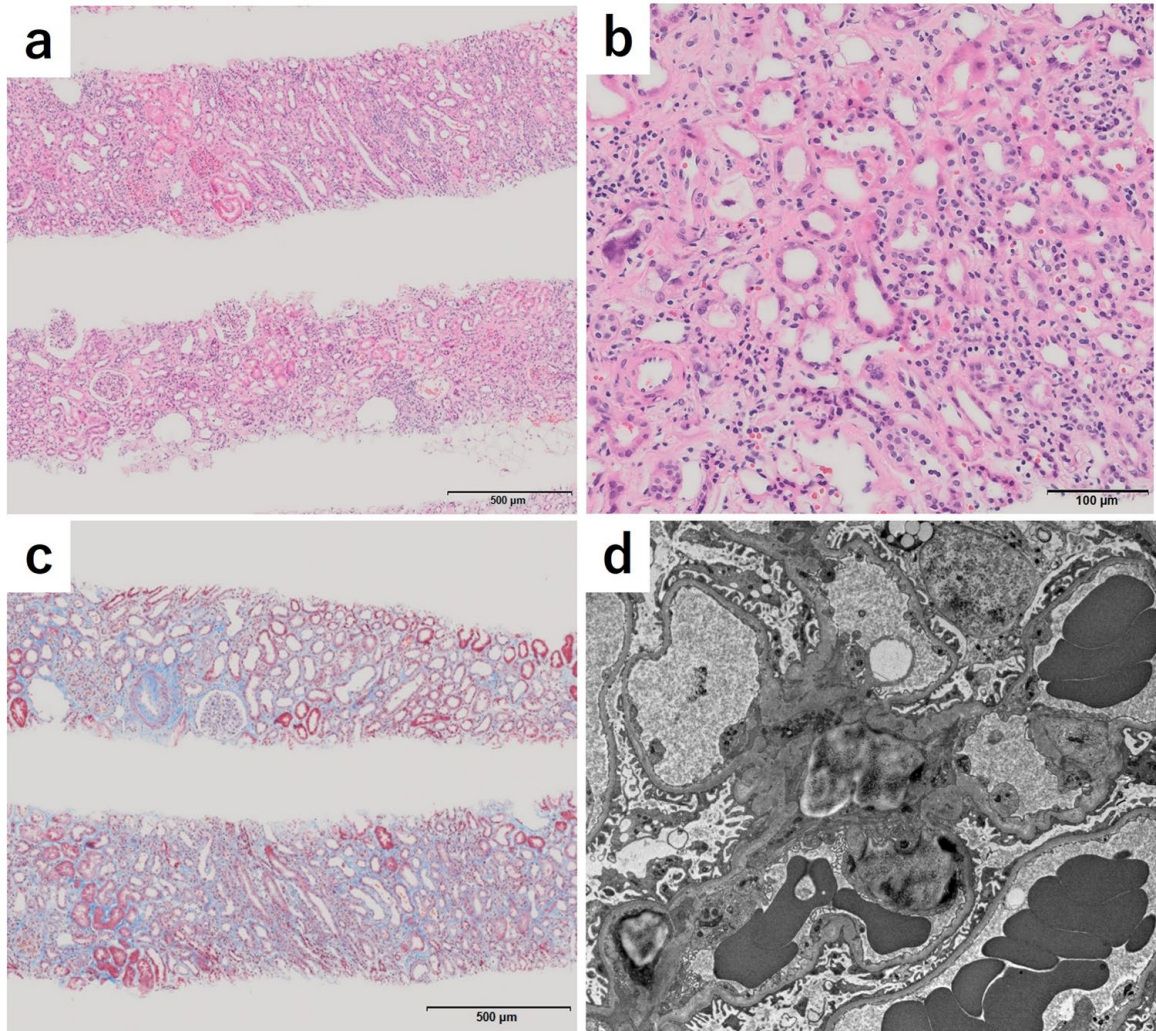
Light microscopic (**a**–**c**) and electron microscopic (**d**) findings of kidney biopsy. **a, b** A mild infiltration of inflammatory cells, chiefly composed of lymphocytes and plasma cells was observed along with a small number of eosinophils in the interstitium of the kidney cortex (hematoxylin–eosin stain). **c** Fibrosis in the tubulointerstitium was mild (Masson trichrome stain). **d** Electron microscopy showed no deposits, no thickening or thinning of the basement membrane, and little foot process effacement in the glomerulus

Upon admission, the patient weight and height were 45.6 kg and 154.5 cm, respectively. The patient’s blood pressure was 132/92 mmHg, pulse rate was 80 beats/min, and body temperature was 36.6 ℃. Chest, heart, and abdominal findings were unremarkable. Ophthalmological examination indicated no uveitis, and no superficial lymphadenopathies or rashes were observed. The electrocardiogram showed no significant abnormal findings. Computed tomography findings did not suggest any post-renal lesions. Table 1 shows the findings of the hospital admission examination. In the urine test, no hematuria was observed, but proteinuria (1.06 g/day) and leukocyturia (10–19 white blood cells/high-power field) were detected. Urinary β2MG and NAG were 126,473 μg/L and 71.7 IU/L, respectively. The white blood cell counts and hemoglobin levels were 3600 cells/μL and 13.0 g/dL, respectively. There were no elevations in laboratory findings suggestive of allergy, such as eosinophils or IgE. The results of blood biochemistry tests indicated that serum Cr was 1.57 mg/dL (estimated glomerular filtration rate, 27.3 mL/min/1.73 m^2^). The levels of electrolyte, liver function tests, and blood glucose were within the normal range. Immunological tests for C-reactive protein, myeloperoxidase antineutrophil cytoplasmic antibody, proteinase 3 antineutrophil cytoplasmic antibody, antinuclear antibody, anti-SS-A/Ro antibodies, and anti-SS-B/La antibodies were all negative. A kidney biopsy was performed to investigate the causes of kidney dysfunction. The sample of kidney cortex contained 44 glomeruli, 1 of which showed global sclerosis, and the remaining 43 glomeruli were almost normal. A mild infiltration of inflammatory cells, chiefly composed of lymphocytes and plasma cells, were observed along with a small number of eosinophils in the interstitium of the kidney cortex (Fig. 1). Tubulitis, tubular atrophy, flattened and expanded proximal tubular epithelium with areas of foamy cytoplasm, and interstitial fibrosis were mildly observed. Immunofluorescent staining for IgG, IgA, IgM, C3, and C1q was all negative. Electron microscopy showed no deposits, no thickening or thinning of the basement membrane, and little foot process effacement in glomerulus. These findings were basically consistent with those typically observed in tubulointerstitial nephritis (TIN).

**Table 1:** Summary of relevant laboratory parameters

Urinalysis			Blood chemistry			Immuno-serology		
Specific gravity	1.036		TP	7.1	g/dL	CRP	0.61	mg/dL
pH	7		Alb	4.1	g/dL	IgG	1079	mg/dL
Protein	1.06	g/day	BUN	23	mg/dL	IgA	215	mg/dL
Occult blood	1+		Cr	1.57	mg/dL	IgM	179	mg/dL
Glucose	3+		UA	1.7	mg/dL	IgE	75	IU/mL
β2MG	126473	μg/gCr	Na	143	mEq/L	C3	179	mg/dL
NAG	71.7	IU/gCr	K	4.3	mEq/L	C4	47	mg/dL
			Cl	107	mEq/L	CH50	86.4	U/mL
Urine sediment			Ca	9.9	mg/dL	ANA	<40×	
RBC	1–4	/HPF	Pi	2.9	mg/dL	Anti-DNA-antibody	<6.0	IU/mL
WBC	10–19	/HPF	AST	19	IU/L	PR3-ANCA	<1.0	U/mL
Cast	Granular	50-99/WF	ALT	19	IU/L	MPO-ANCA	<1.0	U/mL
			γGTP	41	IU/L	anti-SS-A/Ro antibodies	Negative	
			Glu	90	mg/dL	anti-SS-B/La antibodies	Negative	
Hematology								
WBC	10300	/μL						
Hb	13	g/dL						
Plt	46.0×10^4^	/μL						

*β2MG* β2 microglobulin, *NAG* N-acetyl-β-D-glucosaminidase, *RBC* red blood cell, *WBC* white blood cell, *Hb* hemoglobin, *Plt* platelet, *TP* total protein, *Alb* albumin *BUN* blood urea nitrogen, *Cr* creatinine, *UA*, uric acid, *Na* sodium, *K* potassium, *Cl* chlorine, *Ca* calcium, *Pi* phosphorus, *AST* aspartate aminotransferase, *ALT* alanine aminotransferase, *γGTP* γ-glutamyl transpeptidase, *Glu* glucose, *CRP* C-reactive protein, *IgG* immunoglobulin G, *IgA* immunoglobulin A, *IgM* immunoglobulin M, *IgE* immunoglobulin E, *C3* complement c3, *C4* complement c4, *CH50* homolytic complement activity, *ANA* antinuclear antibody, *PR3-ANCA* proteinase-3-antineutrophil cytoplasmic antibody, *MPO-ANCA* myeloperoxidase antineutrophil cytoplasmic antibody

**Fig. 2:**
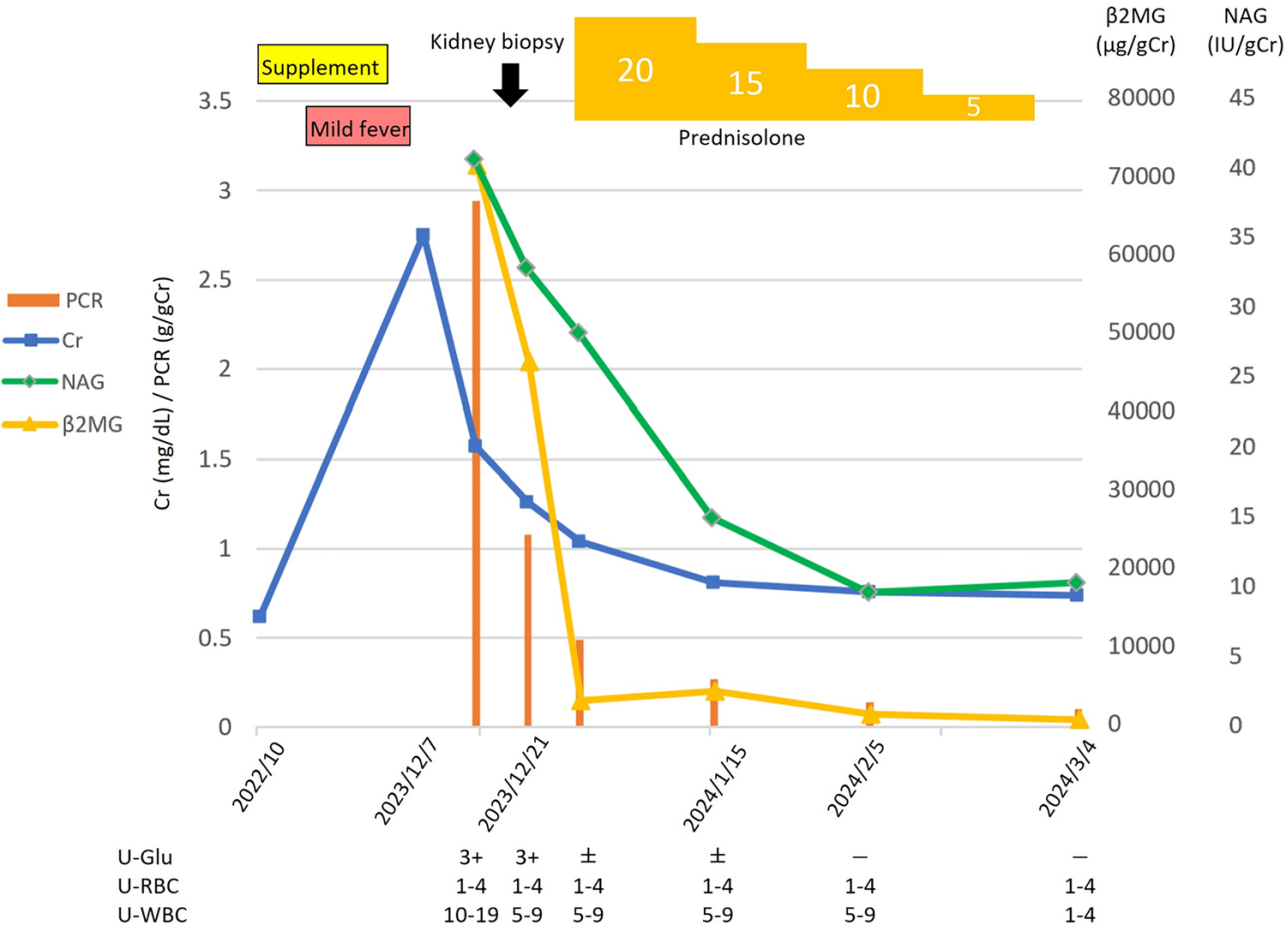
Clinical course of the patient over a period of 3 months, and serum creatinine and urinary NAG, β2MG, PCR (protein/creatinine ratio), glucose, WBC, and RBC are shown

Based ###on a comprehensive consideration of clinical symptoms, blood, urine, and pathological findings, including the absence of fundoscopic abnormalities, we diagnosed acute TIN. Although kidney function tended to improve after discontinuation of Benikoji CholesteHelp^®^, the patient was treated with prednisolone, considering the residual inflammation in the tubulointerstitium. Oral prednisolone was started at a dose of 20 mg/day and tapered by 5 mg/day every 2 weeks until discontinuation. Considering the risk of side effects, the treatment was limited to about 2 months, all kidney impairment markers were improved with the prednisolone treatment, and there was no relapse observed even after the discontinuation of prednisolone (Fig. 2).

## Discussion

Red yeast rice has been used to produce alcoholic beverages and various fermented foods especially in East Asia [1]. It produces a variety of secondary metabolites. Monacolin K has been reported to inhibit cholesterol synthesis in humans [2]. It also produces monacin, a type of azaphyllone pigment reported to improve lipid metabolism and antioxidant effects [3], and citrinin, a fungal toxin that causes nephrotoxicity [4]. Red yeast rice is used worldwide as a health food, but mold toxicity contamination of foods made from red yeast rice has become a problem [5].

Since around March 2024, there have been many cases of kidney dysfunction in people who took certain supplements containing red yeast rice in Japan.

The cause of the renal dysfunction is assumed to be the mold toxin citrinin or some other contaminant, but the details are not clear at this time.

In this case, the pathological findings were similar to those of common tubulointerstitial nephritis, with no specific findings. Fortunately, due to relatively early discontinuation of the supplement after the onset of symptoms, serum creatinine level somewhat improved. However, kidney biopsy findings revealed the infiltration of mild residual inflammatory cell in the interstitium. Therefore, the patient was treated with prednisolone. E Gonza´lez et al. reported that prednisolone should be started promptly after diagnosis of drug-induced TIN to avoid subsequent interstitial fibrosis and an incomplete recovery of renal function [6]. Despite the short intervention, this case indeed showed that kidney function markers recovered to almost normal levels.

It has been reported that Fanconi’s syndrome is often present in cases of renal impairment after taking the same supplement. In the present case, no obvious electrolyte abnormalities diagnostic of Fanconi’s syndrome were found on examination at the time of the initial visit to our hospital, but hypouricemia and urinary sugar were observed. These laboratory abnormalities then mildly resolved over the course of treatment. The patient visited our hospital after voluntarily stopping taking the supplement and may have been in the process of recovering from Fanconi’s syndrome.

As in general drug-induced kidney injury, this case also suggests that the first step in treatment should be discontinuation of the suspected drug. In addition, patients with persistent eGFR decline and elevated tubulointerstitial damage markers may benefit from prednisolone therapy as in other drug-induced kidney disorders. Nevertheless, prednisolone therapy should be carefully evaluated on a case-by-case basis, including the activity of inflammatory findings in the interstitium, since there is a risk of side effects and it is unlikely to be effective in chronic lesions. The symptoms of renal dysfunction typically do not manifest until much later stages, and diagnosis is often delayed. On the other hand, symptoms such as fever and skin rash may occur in the tubulointerstitial nephritis. Although these symptoms may be difficult to distinguish from those of the common disease, early withdrawal of the suspected medication and consultation with a doctor are necessary considering drug-induced kidney damage.

The cause of renal impairment has not yet been identified, but if renal dysfunction is confirmed, it is important to promptly identify and discontinue the drug suspected to be the cause of renal impairment and, if indicated after diagnosis of TIN, steroid therapy should be started immediately.

In conclusion, we have experienced a case of tubulointerstitial nephritis, most likely caused by Benikoji CholesteHelp^®^. For renal damage associated with the use of this supplement, early discontinuation and diagnosis, as well as early treatment including steroids, must be considered.
